# A Stellar Imaging Error Correction Method Based on an Ellipsoid Model: Taking Ziyuan 3-02 Satellite Data Analysis as an Example

**DOI:** 10.3390/s18124259

**Published:** 2018-12-04

**Authors:** Bo Wang, Wei Zhou, Yuyang Gao, Qinghong Sheng

**Affiliations:** College of Astronautics, Nanjing University of Aeronautics and Astronautics, Nanjing 210016, China; zwwei@nuaa.edu.cn (W.Z.); gaoyuyang@nuaa.edu.cn (Y.G.); qhsheng@nuaa.edu.cn (Q.S.)

**Keywords:** star sensor, stellar point imaging error, dynamic frame overlay, ellipsoid model, image star point compensation

## Abstract

Stellar point image coordinates are one of the important observations needed for high-precision space attitude measurement with a star sensor. High-coupling imaging errors occur under dynamic imaging conditions. Using the results of preliminary star point extraction from star sensor imaging data combined with a superimposed time series, we analyze the relative motion and trajectory based on the star point image, establish an image error ellipsoid fitting model based on the elliptical orbit of a satellite platform, and achieve geometric error correction of a star sensors’ image star point using multi-parameter screening of the ambiguous solutions of intersection of the elliptic equations. The simulation data showed that the accuracy of the correction error of this method reached 89.8%, and every star point coordinate required 0.259 s to calculate, on average. In addition, it was applied to real data from the satellite Ziyuan 3-02 to carry out the correction of the star points. The experiment shows that the mean of attitude quaternion errors for all its components was reduced by 52.3%. Our results show that the estimation parameters of dynamic imaging errors can effectively compensate for the star point image observation value and improve the accuracy of attitude calculation.

## 1. Introduction

A star sensor is an electronic measurement system that can measure the three-axis attitude of the carrier satellite using a star as the measurement object and a photodetector as the core component. Owing to their strong autonomy, good concealment, high reliability, and high precision, star sensors have become important instruments for measuring spacecraft attitude in the aerospace field. They are widely used with Earth observation satellites and space exploration aircraft. In recent years, with the rapid development of aerospace science and technology, the dynamic high-precision attitude measurement of aircraft has become even more important for space target surveillance and geosynchronous observation of constellations of spacecraft [1,2,3,4].

Dynamic high-precision attitude measurement has become indispensable for aircrafts [5,6]. Because this technique requires the image to provide extremely accurate star point coordinates, research on resolving errors related to star sensor imaging errors is always ongoing [7]. A star sensor will introduce complex imaging errors in the dynamic imaging process, making the accuracy of attitude measurement difficult to guarantee [8,9]. These imaging errors are due to the relative motion between the starry sky and the camera [10]; star point positioning errors include both static [8,11] and dynamic systematic errors [12]. All errors can cause the star point position to be inaccurate, thus affecting the measured attitude of the satellite platform.

The development of methods for correcting these errors is ongoing. On the theoretical level, Jia et al. [13] used the frequency domain method to explore the factors influencing errors involved in star point extraction from the perspective of an imaging model. However, that study did not shed light on the inherent mechanism of error generation. Yao et al. [14] established a distortion model based on point coordinate measurement, which implements non-uniformity error correction for each pixel. Li et al. [15] pointed out that suppressing pixel non-uniformity noise can greatly improve the accuracy of star point positioning based on analysis of a star point positioning error model. Schmidt et al. [16] considered the in-orbit usage and real-time requirements of the star sensor, treated all noise as pixel non-uniformity noise, predicted the pixel-level noise, and corrected the star point positioning error using the background difference method. Liao et al. [17] considered combining a star sensor and an inertial platform to counteract the negative effects of excessive angular velocity of the carrier satellite and to ensure that star point positioning eliminates cumulative errors. At a practical engineering level, Samaan et al. [18] considered selecting more sensitive chips to avoid star blur while Yang et al. [19] used the least square support vector regression method to train and fit an image to compensate for the image of the square star coordinates. Most of these methods analyzed the star sensor imaging errors from the perspective of imaging principles or hardware and compensated for the errors. The Kalman filter [20] or smoother techniques can improve attitude measurements, but they have high coupling with hardware design model, and are acting on the attitude directly. 

The above studies make important contributions to the theoretical development and application of measurement technology. However, all of them consider one type of error to the neglect of others. In addition, they lack error analysis for star maps obtained from a moving satellite platform. In response, a mathematical model that considers multiple imaging errors needs to be established. In this paper, we develop a generic model by analyzing a large amount of star map data to be used in real-time attitude determination. Specifically, we think about correcting error from the aspect of the data. We describe our aims and demonstrate the effectiveness of our method. Our method is easier to implement than a stricter model, as in our method we do not need to understand how a specific star sensor works. The only thing we need to know is the coordinate before the correction. This powerful model can be used to correct star point coordinates from the same star sensor.

In our method, we consider the star maps of the star sensor to be superimposed, and the relative motion trajectory of the star indicates the motion of the satellite to some extent. We select point coordinates for every trajectory randomly and equably. Then, we conduct bidirectional fitting of the motion trajectory. If the errors obtained by fitting from the two directions are not consistent, a more quadratic fit is required. The fit establishes a link between image position and first coefficients. The image position can be indicated by one set of the point coordinates mentioned as above. The selection principle is that the point coordinates can best match the results of the first fitting. And then these parameters are utilized to restrain and screen the corrected coordinates, so as to improve the accuracy of the corrected coordinates.

This study is based on image data collected by the star sensor. We take the motion of the satellite platform, use an elliptic equation to fit the relative motion trajectory of the star image, and implement geometric error correction of the image star points using multiple parameters to classify the ambiguous solutions of the intersection of the elliptic equation. As long as a set of identical star sensor data are available to obtain the initial model, the coordinates of the star point can be quickly compensated, and the model can be continuously updated to improve its accuracy. Our research shows that after dynamic imaging error analysis and parameter estimation, the intersection of multi-estimated parameter curves can effectively be used to compensate for the image star point observation values. The time required for the method is short, which will allow for better observations for the strict calibration and attitude calculation of subsequent star sensors.

## 2. Methods

### 2.1. Technical Outline of the Ellipsoid Model Method

As mentioned above, we wanted to solve the imaging error problem of the star sensor based on star maps. We considered the star maps of the star sensor to be superimposed, and the relative motion trajectory of the star indicates the motion of the satellite to some extent. Then, we conducted bidirectional fitting of the motion trajectory. Based on this fitting, an elliptic model was established, the parameters were estimated and the coordinates were compensated to correct the star point error.

**Figure 1:**
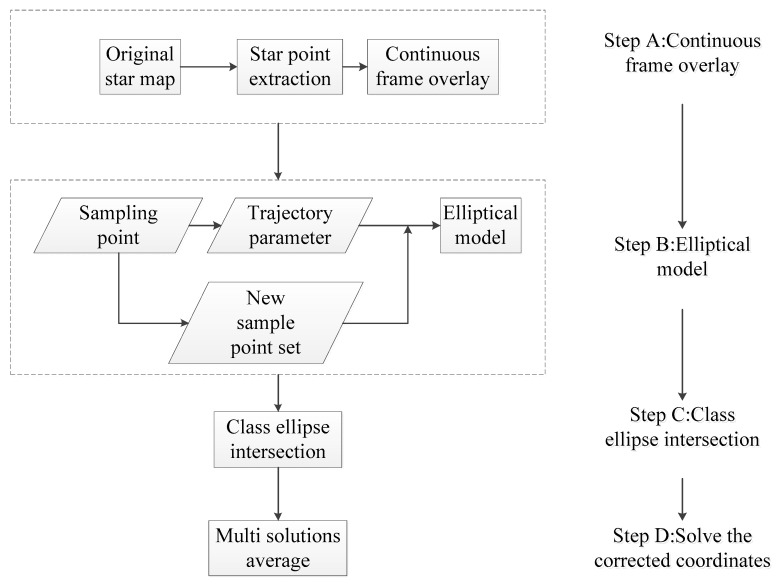
The flow chart of the ellipsoid model method.

Figure 1 shows the flow of the ellipsoid model method. 

### 2.2. Ellipsoid Model of Image Star Points

**Figure 2:**
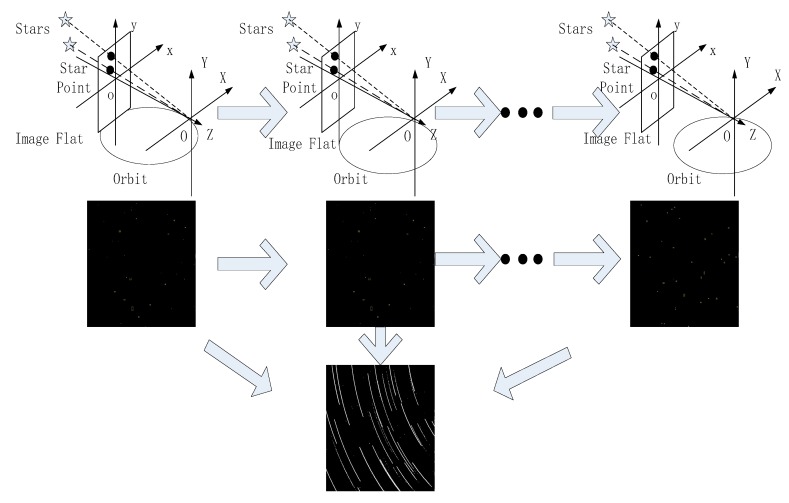
Schematic diagram of the ellipsoid model.

In the celestial system, stars stand at very great distances from satellite platforms that orbit the Earth; therefore, stars can be thought of as relatively static control points that can be used to determine the attitude of a satellite platform. The process of star sensor imaging is shown in Figure 2. During a known period, a satellite moves in an elliptical orbit. Note that a spacecraft experiencing acceleration in any direction will not follow an elliptical orbit. Fortunately, observation satellites are usually not accelerating when observations occur. We consider that when an observation satellite is in a stable attitude, the satellite is slewing at a constant angular rate. As the satellite moves along its elliptical orbit, the camera center of the star sensor changes accordingly. Because the star is stationary, the imaging trajectory of the star during this time reflects the elliptical orbit of the satellite platform to some extent.

However, because the *x* and *y* coordinates in the image are perpendicular to each other and measured relatively independently, the long and short half axes of the elliptical orbit are negligible relative to the star distance during the star sensor imaging process. A single imaging trajectory has two possible directions (the *x* and *y* directions); such polysemy solutions will be considered in subsequent parameter estimation. If the errors obtained by fitting from the two directions are consistent, that is, the errors by fitting from direction *x* are approximately equal to those from direction *y*, then the error can be compensated. If they are not consistent, a more quadratic fit is required.

From the perspective of the image, the imaging trajectory of a single star point can be represented by a certain elliptical arc equation. Since the satellite platform only runs a small elliptical arc orbit during this period, a parabolic equation approximation is used instead of a small elliptical arc equation. This is bidirectionally analyzed from *x* and *y*, respectively, so an image point trajectory equation can be established (Equation (1)).

(1) $$\left\{ \begin{array}{l} x=ay^{2}+by+c\\ y=a\prime x^{2}+b\prime x+c\prime \end{array}\right.$$

In the Equation (1), x,y are a random and uniform selection of points on the relative trajectory of stars after the superposition of multi-frame star maps.

Each type of imaging trajectory in the figure can be fitted with an ellipsoid shape. Now, although the matching ellipse corresponding to each imaging trajectory is not the same, these trajectories are all obtained from the same star maps and are formed by the same satellite platform, so they should have the same regularity. In other words, they can express the motion trajectory of the satellite platform, but some kind of scaling relationship does exist. Therefore, the elliptic coefficient parameter should satisfy a certain model and is related to the image position. The image position can be indicated by one set of the point coordinates x,y in Equation (1). The selection principle is that the selected set of star coordinates should be able to best express the fitting coefficients ($a,b,c,a^{\prime },b^{\prime },c^{\prime }$). That means the point coordinates $\bar{x},\bar{y}$ we selected can best match the results of the first fitting. A quadratic model is used, and its mathematical expression can be expressed as:

(2) $$\left\{ \begin{array}{l} a=k_{1}{\bar{x}}^{2}+m_{1}{\bar{y}}^{2}+n_{1}\bar{x}\bar{y}+p_{1}\bar{x}+q_{1}\bar{y}+l_{1}\\ b=k_{2}{\bar{x}}^{2}+m_{2}{\bar{y}}^{2}+n_{2}\bar{x}\bar{y}+p_{2}\bar{x}+q_{2}\bar{y}+l_{2}\\ c=k_{3}{\bar{x}}^{2}+m_{3}{\bar{y}}^{2}+n_{3}\bar{x}\bar{y}+p_{3}\bar{x}+q_{3}\bar{y}+l_{3}\\ a^{\prime }=k_{4}{\bar{x}}^{2}+m_{4}{\bar{y}}^{2}+n_{4}\bar{x}\bar{y}+p_{4}\bar{x}+q_{4}\bar{y}+l_{4}\\ b^{\prime }=k_{5}{\bar{x}}^{2}+m_{5}{\bar{y}}^{2}+n_{5}\bar{x}\bar{y}+p_{5}\bar{x}+q_{5}\bar{y}+l_{5}\\ c^{\prime }=k_{6}{\bar{x}}^{2}+m_{6}{\bar{y}}^{2}+n_{6}\bar{x}\bar{y}+p_{6}\bar{x}+q_{6}\bar{y}+l_{6} \end{array}\right.$$

In this equation, $\bar{x},\bar{y}$ are the image coordinates, which can best match the results of the first fitting, a, b, c, $a^{\prime }$, $b^{\prime }$, and $c^{\prime }$ are the fitting coefficients of the equations in Equation (1), k, m, n, p, q and l are the fitting coefficients, and thus, as Equation (2), the equation is called the ellipsoid model.

### 2.3. Parameter Estimation and Coordinate Compensation

In the above process, the star map data acquired by the star sensor is expressed by a set of parameterized elliptic equations, and the actual imaging error has been smoothed and corrected in the fitting process of the superimposed image traces. Using the elliptic equations of the above six related parameters, we can realize the correction and compensation of the image observations before calibration and attitude calculation of the star sensor.

**Figure 3:**
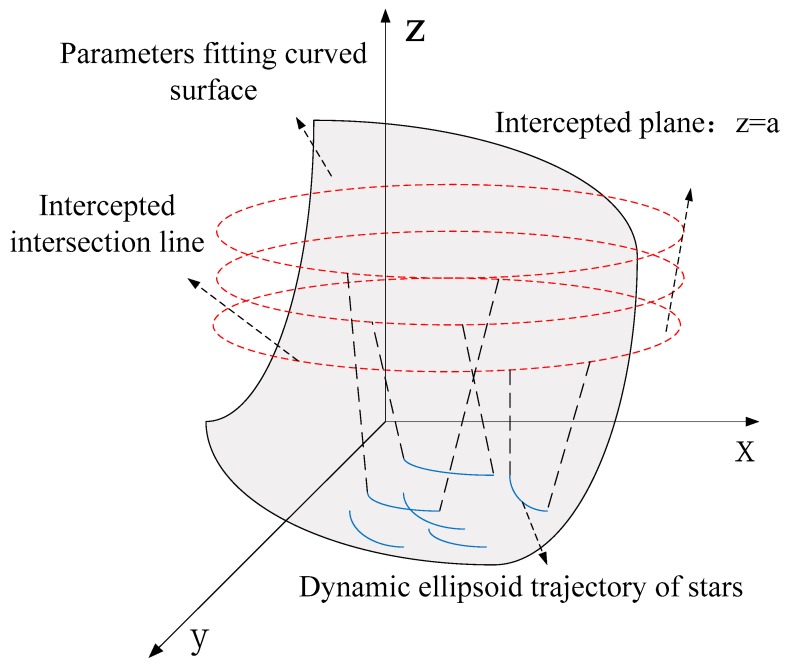
Principle of the ellipsoid model.

The principle on which the ellipsoid model is based shows that any trajectory in the image plane will correspond to a curve on the surface (Figure 3). The specific principle is that the parameter value is high, parallel to the *xoy* plane, the intersection of the plane is the corresponding conic, and solutions can be found by intersecting the conic of each group. In theory, the intersection point is represented by the corrected coordinates obtained.

**Figure 4:**
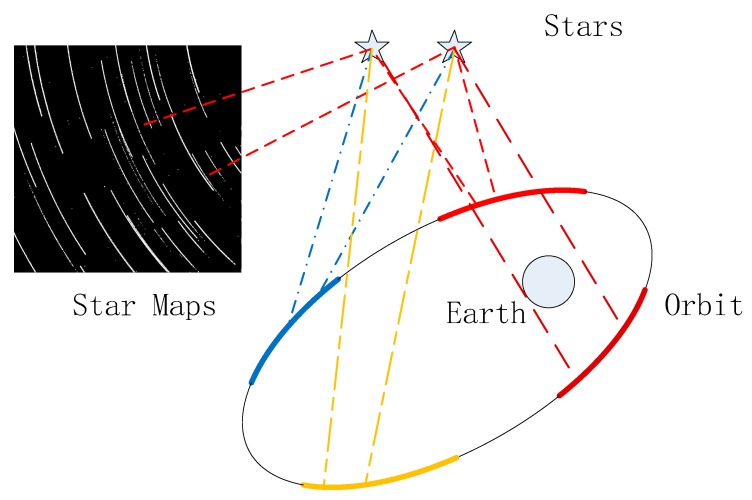
Reasons for ambiguous solutions.

But actually, in Figure 4 the upper left corner provides a superimposed imaging trajectory of multi-frame data. The black figure in the upper left corner results from superimposing multi-frame consequent star maps. We can get many relative motion trajectories, as shown by the white curve. Because the star is stationary, the trajectory corresponds to the regular orbit of the spacecraft. The ellipse in this figure illustrates the orbit and the motion of the satellite platform; the circle at one focus represents the earth. According to the analysis, the traces after multi-frame data superposition are the embodiment of the orbit equation of the satellite platform. Although different stars have different imaging positions, these traces over a specific period should conform to an arc of the elliptical orbit, which is the motion trajectory represented by the arcs (shown in four colors). The real meaning of the four arcs is the orbit of the spacecraft, which is a schematic diagram, meaning the traces in a specific period should confirm to the ellipsoid. and the motion trajectory we got may correspond random one of them. 

This explains why the results have an ambiguous solution. At this time, it is theoretically reasonable and effective to use an elliptic equation to fit the imaging error on the image path. Because it is a conic shape, the intersection solution will have an ambiguous solution meaning it must be filtered.

**Figure 5:**
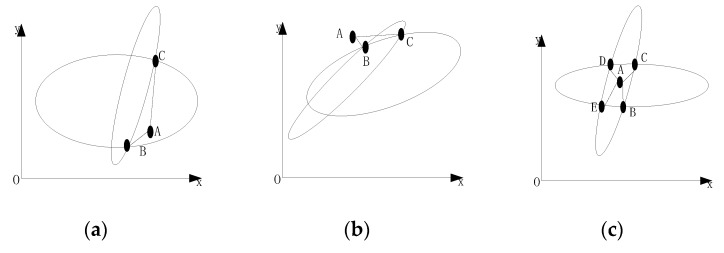
Principle of filtering ambiguous solutions: (**a**) coordinate *x* of ambiguous solutions closer to original star point; (**b**) coordinate *y* of ambiguous solutions closer to original star point; (**c**) all ambiguous solutions almost close to original star point.

We now describe the principle involved in intersecting ambiguous solution filtering. As shown in Figure 5a,b, point A is the original star point; one coordinate of point C is closer to point A, but the other coordinate is farther away. Therefore, point B the closer point should be selected as the candidate point in the modified solution. As shown in Figure 5c, four ambiguous solutions of point BCDE are possible. Therefore, it is necessary to consider the two-way coordinates comprehensively and select the point closest to point A as the candidate point of the modified solution.

**Figure 6:**
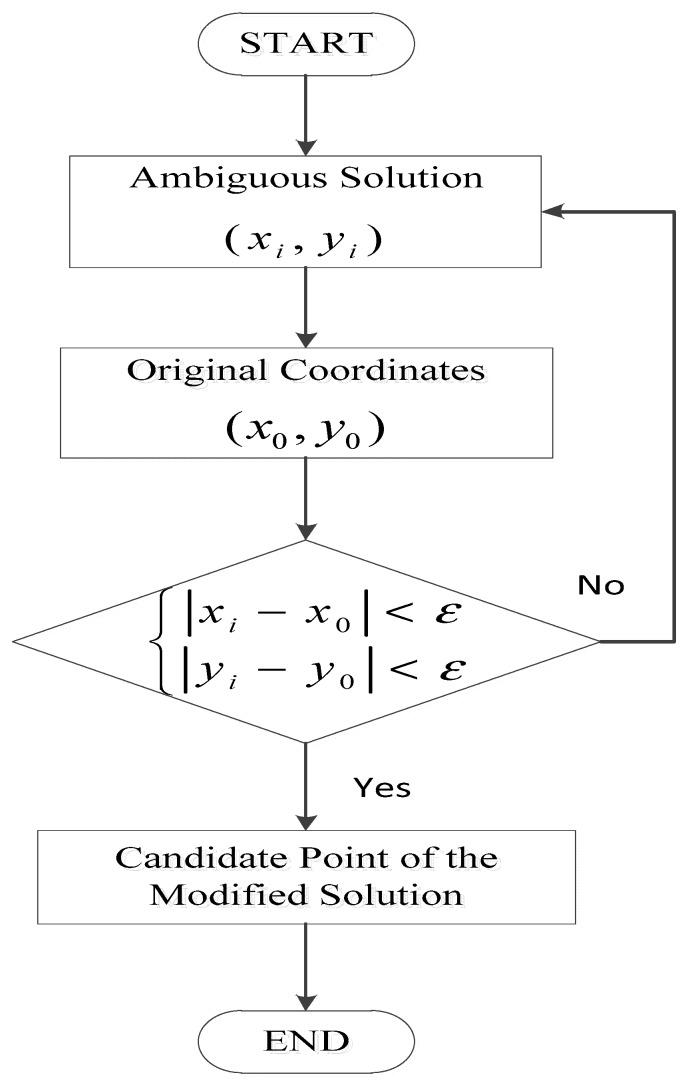
Flow chart of filtering ambiguous solutions.

Figure 6 shows the steps for filtering the ambiguous solution. Here, we set a value ε to filter the ambiguous solution; meets this value, we will call it candidate point of the modified solution. In the simulation experiment, the value is set as 2 pixels, while in the real data experiment, the value was set as 15 pixels.

## 3. Experiment and Results Analysis

### 3.1. Experiment Data

In this paper, two sets of experiments were designed, one simulated and one using for the real data.

The design idea of the simulation experiment is as follows: first, we simulated a set of original star maps using MATLAB2014 and added Gaussian white noise. We then carried out rough extraction of the star points. Next, the ellipsoid model was applied to the dataset to correct the coordinates of the star point. Here, we assumed that the simulated star point coordinates before the noise was added were the real coordinates; the corrected coordinates were compared with the real coordinates.

The simulation conditions were as follows: Something about the computer: frequency of the CPU was 1.70 GHz, memory was 4.00 GB, system was Windows 8.1. We input the initial a set of attitudes of satellites, and then simulated using the principles of geometric imaging. In this process, we assumed that the focal length of the camera was f = 43.3 mm, the pixel size was 0.015 mm, the signal-to-noise ratio (SNR) was 5 dB, the star’s star limit was set to 6, and the photo size was set to 1024 × 1024.

In the second set of experiments, the ellipsoid model was applied to the real data to correct the star point error. Then the satellite attitude was determined. In this paper, the satellite attitude was represented by quaternions, and compared with the quaternions provided by the original gyroscope.

The experimental data used in this experiment are all real data. obtained by the star sensor aboard the carrier satellite ZY-3-02 of China. Two sets of data were used: the group 0702 star maps was obtained on 2 July 2016, and the group 0712 was obtained on 12 July 2016. The size of each star map was 1024 × 1024 pixel. The group 0702 was used as raw data for model fitting and the group 0712 was used to test the model when conducting the comparison test. First, the 0702 data were used to test the conformity of the model to its own data to prove the correction of the model. Then the 0712 data were used to test the applicability and effectiveness of the model. 

In reference to the correction test of this model, for the simulation experiment, we can assume that the MATLAB star point coordinates were exact before the noise was added, meaning the accuracy of the correction can be directly verified. For the real data, the real coordinates were not clear, so the external precision evaluation could not be performed. The mean square error of the difference between the two was evaluated using the coincidence degree of data.

### 3.2. Experiment Results and Analysis

#### 3.2.1. Experiment Results of Simulation

As described above, the present study began by performing two-way fitting in the *x* and *y* directions of the frame superposition results of simulation data; We select 100 point coordinates of every trajectory and fit the point coordinates. The resulting fitting equations are shown in Equations (3) and (4).

(3) $$\{\begin{array}{c} x_{1}=7.68287111e^{-3}y_{1}^{2}+1.69300874y_{1}+661.87217940\\ x_{2}=2.09244654e^{-3}y_{2}^{2}+0.93951053y_{2}+271.60179426\\ x_{3}=1.51004741e^{-3}y_{3}^{2}+0.83393057y_{3}+127.79101785\\ x_{4}=2.88847604e^{-3}y_{4}^{2}+0.67623070y_{4}+290.02821260\\ x_{5}=1.97606378e^{-3}y_{5}^{2}+0.71818976y_{5}+168.06019984\\ x_{6}=1.73496887e^{-3}y_{6}^{2}+0.41826417y_{6}+3.5860763886 \end{array}$$

(4) $$\{\begin{array}{c} y_{1}=-7.10384860e^{-4}x_{1}^{2}+1.49411085x_{1}-677.38150299\\ y_{2}=-7.71334248e^{-4}x_{2}^{2}+1.33870777x_{2}-302.26592681\\ y_{3}=-8.05331580e^{-4}x_{3}^{2}+1.24388547x_{3}-140.42369637\\ y_{4}=-6.81424112e^{-4}x_{4}^{2}+1.27494096x_{4}-290.11935213\\ y_{5}=-7.35369538e^{-5}x_{5}^{2}+1.23711026x_{5}-170.93902255\\ y_{6}=-6.46955865e^{-4}x_{6}^{2}+1.05814885x_{6}+50.97186378 \end{array}$$

**Figure 7:**
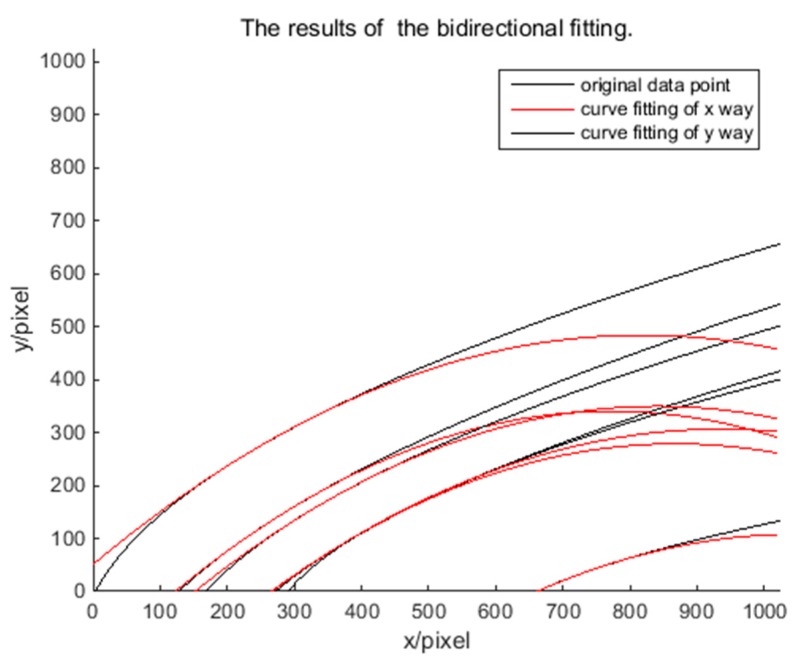
The results of the bidirectional fitting.

**Figure 8:**
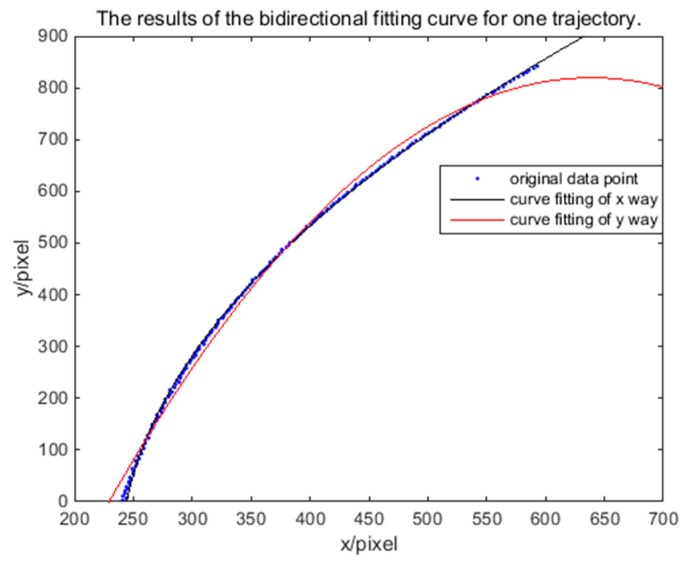
The results of the bidirectional fitting curve for one trajectory.

The results of bidirectional fitting are shown using MATLAB software. Figure 7 shows that the fitting results from the *x* and *y* directions are not very consistent in general. Therefore, starting from a single trajectory, Figure 8 shows the results of the bidirectional fitting curve for the one trajectory. A difference exists in the two-way error; this difference will affect the determination of the error and the correction of the coordinates. After calculation, the average error in the *x* direction of fitting is 1.86 pixels; that in the y direction of fitting is 0.483 pixels. So, if we correct error from one direction, *x* or *y*, the result is not accurate. Therefore, we propose a method that involves using an ellipsoid model and an intersection to correct the error. Specifically, based on the two-way fitting curve, the coefficients are quadratically fitted. In theory, the required coordinate correction solution should satisfy the quadratic fitting equation of each coefficient; however, when considering the actual situation and the existence of the fitting error, the results of the intersection of each quadratic fitting curve were regarded as the required coordinate correction solution. Because the result of each intersection may be a modified solution, each result was recorded as a candidate point of the modified solution; and the average value was used as the final correction solution.

Then, we selected most the suitable point coordinate for every trajectory, so we selected six point coordinates to fit the coefficients, and the results are as follows:

Equations (5) and (6) provide the results of quadratic fitting.

(5) $$\{\begin{array}{c} a=1.6608e^{-7}{\bar{x}}^{2}+2.7379e^{-7}{\bar{y}}^{2}\;+4.5693e^{-7}\bar{x}\bar{y}-2.1240e^{-4}\bar{x}\;-2. 8122e^{-4}\bar{y}+6.8125e^{-2}\\ b=\;-4.9939e^{-5}{\bar{x}}^{2}\;-1.1482e^{-4}{\bar{y}}^{2}-1.8560e^{-4}\bar{x}\bar{y}+7.4011e^{-2}\bar{x}+1.1331e^{-1}\bar{y}-23.778\\ c=3.2112e^{-3}{\bar{x}}^{2}+7.8633e^{-3}{\bar{y}}^{2}+1.0791e^{-2}\bar{x}\bar{y}-3.7043\bar{x}-8.1798\bar{y}+1579.1764 \end{array}$$

(6) $$\{\begin{array}{c} a^{\prime }=1.3153e^{-9}{\bar{x}}^{2}+4.6631e^{-9}{\bar{y}}^{2}+5.4640e^{-9}\bar{x}\bar{y}-1.5831e^{-6}\bar{x}-2.9625e^{-6}\bar{y}-3.5370\\ b^{\prime }=-2.7497e^{-6}{\bar{x}}^{2}-4.3032e^{-6}{\bar{y}}^{2}-7.6637e^{-6}\bar{x}\bar{y}+3.9498e^{-3}\bar{x}+3.3121e^{-3}\bar{y}+0.2368\\ c^{\prime }=1.2293e^{-3}{\bar{x}}^{2}+1.1384e^{-3}{\bar{y}}^{2}+3.1252e^{-3}\bar{x}\bar{y}-2.4382\bar{x}-0.2757\bar{y}+354.3663 \end{array}$$

Table 1 compares the coordinates before and after the correction of the simulation data, and the amount of time needed to correct each coordinate. Table 2 compares the errors before and after the correction of the simulation data. According to the tables we can calculate the mean square error of the errors. After calculation, the mean square error of the errors before correction was 0.4709, and the mean square error after correction was 0.0479, and the accuracy improvement of 89.8%. Besides, the average time required for a star point coordinate correction is 0.259 s. Therefore, the model can correct the star point errors effectively and quickly. From the experimental results, the time required for the model can fully meet the needs of typical missions.

#### 3.2.2. Experiment Results of Real Data

As described above, this study began by performing two-way fitting in the *x* and *y* directions of the frame superposition results; We select 120 point coordinates of every trajectory and fit the point coordinates. The obtained fitting equations are shown in Equations (7) and (8).

(7) $$\{\begin{array}{c} x_{1}=6{.06412528e}^{-4}y_{1}{}^{2}+1{.66896674e}^{-1}y_{1}+796.35949068\\ x_{2}=5{.603967868e}^{-4}y_{2}{}^{2}+1{.51297560e}^{-1}y_{2}+777.4138421\\ x_{3}=6{.94229250e}^{-4}y_{3}{}^{2}+3{.87739417e}^{-2}y_{3}+782.26733260\\ \vdots \\ x_{26}=3{.75154707e}^{-4}y_{26}{}^{2}-1{.52832756e}^{-1}y_{26}-138.7228401 \end{array}$$

Detailed equations see Appendix A Equations (A1)–(A26).

(8) $$\{\begin{array}{c} y_{1}=-8{.45008923e}^{-3}x_{1}{}^{2}+16.94088086x_{1}-8104.997895661\\ y_{2}=-6{.39386799e}^{-3}x_{2}{}^{2}+13.43513064x_{2}-6553.51591621\\ y_{3}=-4{.54951256e}^{-3}x_{3}{}^{2}+10.01200305x_{3}-4939.881501419\\ \vdots \\ y_{26}=-2{.23761618e}^{-3}x_{26}{}^{2}+2.03570683x_{26}+845.427003311 \end{array}$$

For detailed equations see Appendix A Equations (A27)–(A52). 

Then, we selected the most suitable point coordinates for every trajectory, so we select twenty-six point coordinates to fit the coefficients, and the results are as follows:

Equations (9) and (10) provide the results of quadratic fitting.

(9) $$\{\begin{array}{c} a=3{.055e}^{-10}{\bar{x}}^{2}-7{.183e}^{-11}{\bar{y}}^{2}+2{.223e}^{-10}\bar{x}\bar{y}+1{.069e}^{-7}\bar{x}+1{.097e}^{-7}\bar{y}+2{.965e}^{-4}\\ b=5{.749e}^{-9}{\bar{x}}^{2}-2{.552e}^{-7}{\bar{y}}^{2}-7{.113e}^{-7}\bar{x}\bar{y}+1{.209e}^{-4}\bar{x}+1{.588e}^{-5}\bar{y}+9{.857e}^{-2}\\ c=-4{.636e}^{-5}{\bar{x}}^{2}+3{.324e}^{-5}{\bar{y}}^{2}+4{.137e}^{-5}\bar{x}\bar{y}+1.023\bar{x}-0.247\bar{y}+9.633 \end{array}$$

(10) $$\{\begin{array}{c} a\prime =-5{.034e}^{-9}{\bar{x}}^{2}-1{.285e}^{-8}{\bar{y}}^{2}-5{.001e}^{-11}\bar{x}\bar{y}+4{.153e}^{-6}\bar{x}+1{.839e}^{-5}\bar{y}-8{.775e}^{-3}\\ b\prime =8{.300e}^{-6}{\bar{x}}^{2}+1{.222e}^{-5}{\bar{y}}^{2}-1{.277e}^{-5}\bar{x}\bar{y}+4{.970e}^{-3}\bar{x}-1{.503e}^{-2}\bar{y}+6.963\\ c\prime =-5{.794e}^{-3}{\bar{x}}^{2}-3{.412e}^{-3}{\bar{y}}^{2}+8{.521e}^{-3}\bar{x}\bar{y}-4.190\bar{x}+3.772\bar{y}-469.338 \end{array}$$

For detailed equations see Appendix A Equations (A53) and (A54). 

Table 3 and Table 4 compare the coordinates before and after the correction of the 0702 and 0712 groups, respectively, using the ellipsoid model. Table 3 and Table 4 represent two different experimental datasets. The entries in the table respectively refer to the horizontal pixel coordinate of points before the correction, the vertical coordinate before the correction, the horizontal pixel coordinate of points after the correction, the vertical coordinate after the correction, the difference between the horizontal pixel coordinate of points before and after the correction, the difference between the vertical coordinate of points before and after the correction, and the time required to correct each coordinate.

Table 3 and Table 4 show that most of the differences for the 0702 group correction are at the pixel level; meanwhile, for the 0712 group, the differences involve more than a dozen pixels. After calculation, the average time to calculate a star point coordinate of the 0702 group is 0.272 s. However, for the 0712 group 0.284 s were required. We know that the correction effect of the ellipsoid model for the 0702 group was significantly better than that of the 0712 group.

Table 5 gives the mean square error of the difference between the two groups of experiments. We can see the model has more complexity than the 0712 group data and the correction effect has improved. We can conclude that the model not only applies effectively to its own data, but also can apply to other data. This shows that the model has good correction ability, applicability, and effectiveness.

In addition, we used the coordinates before and after the correction to calculate the attitude. The attitude directly obtained by gyroscope was used as the original reference attitude. The quaternion was used to measure the accuracy comparison before and after the correction. Table 6 gives a comparison of the quaternion calculated before and after the correction with the quaternion provided by the gyroscope. Table 7 shows the mean of the errors of the four components before and after the correction, and the mean of the combined error. From the table, we can see that the error obtained after correction is reduced by 52.3%, demonstrating the validity of the method.

## 4. Discussion

This paper describes a model of the pixel coordinates of the star point centroid during attitude measurement by a star sensor. Although the pixel coordinates of the star can be given more accurately, some problems still exist. First, because a motion streak has a certain width in the star map after the frame superposition, when we did the quadratic curve fitting, we had to select random points as uniformly as possible within the surface of a certain area before the center curve was fitted. Thus, the accuracy of the model was related to the measured points. In addition, when the second fitting was performed, the number of selected points was small, resulting in the low model conformity. A data source needed to be added to solve this problem. Another weakness is that the model was established on the assumption of stable satellite attitude; if the spacecraft was accelerating or decelerating, its applicability was limited. We will address this shortcoming in our future work. 

## 5. Conclusions

Based on simulation data and real-life imaging data from a star sensor on ZY-3-02, this paper proposes a star point centroid determination model of the whole star map based on the trajectory of motion and the correlation between stars in the same star domain. In simulation experiments, we can calculate the mean square error of the errors. In the example herein, the mean square error of the errors before correction is 0.4709, and the mean square error after correction is 0.0479, an improvement in accuracy of 89.8%. Besides, the average time required for a star point coordinate correction is 0.259 s. Therefore, the model can correct the star point error effectively and the time required for the model can fully meet the needs of typical missions. In real data experiments, we can see that the error obtained after correction is reduced by 52.3%, demonstrating validity of this method for star point coordinate correction What is more, the 0702 group and the 0712 group data express that the model not only applies effectively to its own data, but also can apply to other data. This shows that the model has strong correction ability, applicability, and effectiveness. 

As the number of current data points is small, the correction effect is not very good. However, herein, we prosed a new method for star point centroid correction, proved the applicability and effectiveness of the method, and provided a strong correlated star point centroid and image control point coordinates for subsequent star map recognition. Our work should help with star map recognition and determination of the initial attitude of the satellite.

## Figures and Tables

**Table 1:** The coordinate comparison table before and after the correction of simulation data.

Real Coordinate $x_{0}/\mathrm{Pixel}$	Real Coordinate $y_{0}/\mathrm{Pixel}$	Coordinate before the Correction $x_{1}/\mathrm{Pixel}$	Coordinate before the Correction $y_{1}/\mathrm{Pixel}$	Coordinate after the Correction $x_{2}/\mathrm{Pixel}$	Coordinate after the Correction $y_{2}/\mathrm{Pixel}$	Time of Correction/s
769.5230	51.6882	769.4922	51.6136	769.9165	51.7856	0.267
428.8964	129.9844	429.6600	128.5301	428.4559	129.7142	0.253
756.1361	143.6791	756.4673	143.0625	755.8765	143.6954	0.255
324.2521	178.2613	325.0366	178.8114	323.7439	178.6738	0.254
506.1756	180.5237	507.7108	180.8036	505.8419	180.8086	0.260
344.5397	167.9456	342.6581	168.2032	343.6593	168.0500	0.263

**Table 2:** The mean square error of errors table before and after the correction of simulation.

Errors before the correction/pixel	0.0808	1.6426	0.6999	0.9581	1.5605	1.8992
Errors after the correction/pixel	0.4054	0.5166	0.2601	0.6546	0.4388	0.8866

**Table 3:** The coordinate comparison table before and after the correction of the 0702 groups.

Numbers	Coordinate before the Correction $x_{0}/\mathrm{Pixel}$	Coordinate before the Correction $y_{0}/\mathrm{Pixel}$	Coordinate after the Correction $x_{1}/\mathrm{Pixel}$	Coordinate after the Correction $y_{1}/\mathrm{Pixel}$	D−Value Δx/Pixel	D−Value Δy/Pixel	Time of Correction/s
1	796	86	796.9949	85.18099	−0.9949	0.819	0.285
2	697	260	697.7184	258.9675	−0.7184	1.0325	0.288
3	812	530	815.7728	531.539	−3.7728	−1.539	0.269
4	676	301	676.0389	300.9522	−0.0389	0.0478	0.263
5	651	336	650.5735	336.4726	0.4265	−0.4726	0.243
6	550	348	549.5405	348.5098	0.4595	−0.5098	0.264
7	676	603	676.9681	603.761	−0.9681	−0.761	0.274
8	672	626	674.1938	624.0945	−2.1938	1.9055	0.272
9	658	646	658.367	646.5949	−0.367	−0.5949	0.271
10	442	276	443.7777	274.1592	−1.7777	1.8408	0.268
11	621	790	618.8719	787.6551	2.1281	2.3449	0.278
12	271	164	270.568	165.9817	0.432	−1.9817	0.273
13	250	153	250.4332	153.4049	−0.4332	−0.4049	0.266
14	116	147	114.5381	148.6052	1.4619	−1.6052	0.284
15	82	158	81.45622	156.7154	0.5438	1.2846	0.276

**Table 4:** The coordinate comparison table before and after the correction of the 0712 groups.

Numbers	Coordinate before the Correction $x_{0}/\mathrm{Pixel}$	Coordinate before the Correction $y_{0}/\mathrm{Pixel}$	Coordinate after the Correction $x_{1}/\mathrm{Pixel}$	Coordinate after the Correction $y_{1}/\mathrm{Pixel}$	D-Value Δx/Pixel	D-Value Δy/Pixel	Time of Correction/s
1	701	243	703.9327	239.6972	2.9327	−3.3028	0.287
2	699	247	700.9408	244.4221	1.9408	−2.5779	0.263
3	706	251	704.8268	251.6018	−1.1732	0.6018	0.284
4	703	255	702.3503	255.2876	−0.6497	0.2876	0.298
5	707	258	703.726	260.3408	−3.274	2.3408	0.276
6	705	262	702.381	264.8682	−2.619	2.8682	0.283
7	703	250	703.6072	249.3701	0.6072	−0.6299	0.279
8	702	266	699.179	267.7982	−2.821	1.7982	0.294
9	709	269	702.1712	273.5862	−6.8288	4.5862	0.278
10	704	273	699.3447	277.8297	−4.6553	4.8297	0.298
11	710	276	701.3928	282.0021	−8.6072	6.0021	0.270
12	706	279	697.5668	285.7076	−8.4332	6.7076	0.282
13	711	282	706.9911	293.0142	−4.0089	11.0142	0.278
14	709	285	701.2951	293.4709	−7.7049	8.4709	0.272
15	707	290	697.0002	299.0767	−9.9998	9.0767	0.297
16	712	292	700.0789	303.2288	−11.9211	11.2288	0.294
17	709	299	698.7398	308.9167	−10.2602	9.9167	0.289
18	708	295	703.9664	306.281	−4.0336	11.281	0.296
19	711	303	700.7709	315.0803	−10.2291	12.0803	0.277
20	716	306	698.7524	321.6062	−17.2476	15.6062	0.286

**Table 5:** The mean square error of the difference between the two groups.

Groups	Lateral Mean Square Error $\sigma_{x}/\mathrm{Pixel}$	Longitudinal Mean Square Error $\sigma_{y}/\mathrm{Pixel}$
0702	1.4702	1.3225
0712	5.0044	5.2348

**Table 6:** Comparison of quaternion of the 0712 group.

Real Quaternion (q0, q1, q2, q3)	Quaternion before the Correction (q0, q1, q2, q3)	Quaternion after the Correction (q0, q1, q2, q3)
(0.6865, −0.0656, 0.7057, 0.1623)	(0.6882, 0.1548, 0.6928, 0.1498)	(0.7029, −0.0632, 0.7050, 0.0697)
(0.6865, −0.0658, 0.7058, 0.1621)	(0.6885, 0.1535, 0.6931, 0.1485)	(0.7028, −0.0644, 0.7049, 0.0710)
(0.6864, −0.0659, 0.7059,0.1619)	(0.6883, 0.1546, 0.6928, 0.1495)	(0.7038, −0.0537, 0.7058, 0.0602)
(0.6865, −0.0658, 0.7058, 0.1621)	(0.6881, 0.1556, 0.6925, 0.1506)	(0.7042, −0.0481, 0.7062, 0.0546)

**Table 7:** Comparison of mean of errors of quaternion.

	Mean of q0 Error	Mean of q1 Error	Mean of q2 Error	Mean of q3 Error	Mean of All
Mean of quaternion errors before the correction	0.0018	0.2204	0.0130	0.0125	0.0619
Mean of quaternion errors after the correction	0.0169	0.0024	0.0003	0.0982	0.0295

## Appendix A

As described above, the present study began by performing bidirectional fitting in the *x* and *y* directions of the frame superposition results of 0702 group; the obtained fitting equations are shown as follows:

Equations (A1)–(A26):

(A1) $$x_{1}=6{.064125277003e}^{-4}y_{1}{}^{2}+1{.6689667394e}^{-1}y_{1}+796.3594906760$$

(A2) $$x_{2}=5{.603967862245e}^{-4}y_{2}{}^{2}+1{.5129755955e}^{-1}y_{2}+777.4138421293$$

(A3) $$x_{3}=6{.942292495556e}^{-4}y_{3}{}^{2}+3{.8773941718e}^{-2}y_{3}+782.2673326060$$

(A4) $$x_{4}=6{.9866696267418e}^{-4}y_{4}^{2}+7{.3468440479e}^{-2}y_{4}+636.9685887266$$

(A5) $$x_{5}=6{.92550589668876e}^{-4}y_{5}^{2}-1{.108955567e}^{-1}y_{5}+675.5242865971$$

(A6) $$x_{6}=5{.32516700176634e}^{-4}y_{6}{}^{2}+9{.79781524e}^{-2}y_{6}+598.1488366720$$

(A7) $$x_{7}=5{.26405122100068e}^{-4}y_{7}{}^{2}+7{.032782218e}^{-2}y_{7}+567.6751982558$$

(A8) $$x_{8}=4{.94957822979376e}^{-4}y_{8}{}^{2}+7{.706918615e}^{-2}y_{8}+522.9766827270$$

(A9) $$x_{9}=4{.54097927642127e}^{-4}y_{9}{}^{2}+9{.896728706e}^{-2}y_{9}+460.0217822490$$

(A10) $$x_{10}=5{.15730007995388e}^{-4}y_{10}{}^{2}+2{.69593363783739e}^{-3}y_{10}+486.5398225760$$

(A11) $$x_{11}=6{.53122667389336e}^{-4}y_{11}{}^{2}-2{.50385151027044e}^{-1}y_{11}+572.6263772800$$

(A12) $$x_{12}=5{.85490588013185e}^{-4}y_{12}{}^{2}-1{.42415989149641e}^{-1}y_{12}+505.8106528400$$

(A13) $$x_{13}=4{.74126270204956e}^{-4}y_{13}{}^{2}+3{.83482088667469e}^{-2}y_{13}+392.2837162508$$

(A14) $$x_{14}=4{.71203052395599e}^{-4}y_{14}{}^{2}-3{.31811303106649e}^{-2}y_{14}+354.4012201753$$

(A15) $$x_{15}=3{.79915059769796e}^{-4}y_{15}{}^{2}+8{.98617759975948e}^{-2}y_{15}+244.3665492481$$

(A16) $$x_{16}=3{.24462995469601e}^{-4}y_{16}{}^{2}+1{.52415000053149e}^{-1}y_{16}+218.4113406868$$

(A17) $$x_{17}=5{.55508684171826e}^{-4}y_{17}{}^{2}-2{.41104822702083e}^{-1}y_{17}+358.3023588778$$

(A18) $$x_{18}=4{.64037170393650e}^{-4}y_{18}{}^{2}-1{.48369917015442e}^{-1}y_{18}+208.5281832245$$

(A19) $$x_{19}=3{.33949002276114e}^{-4}y_{19}{}^{2}+9.{38688532562692e}^{-2}y_{19}+95.7945289242\;$$

(A20) $$x_{20}=3{.15382117297735e}^{-4}y_{20}{}^{2}+1{.13261214115208e}^{-1}y_{20}+56.4667047992$$

(A21) $$x_{21}=3{.49587932089585e}^{-4}y_{21}{}^{2}+3{.76491335780276e}^{-3}y_{21}+8.7743658633\;$$

(A22) $$x_{22}=3{.41376891070825e}^{-4}y_{22}{}^{2}+2{.62880465034960e}^{-2}y_{22}-56.0527664244\;$$

(A23) $$x_{23}=3{.47137228707709e}^{-4}y_{23}{}^{2}-2{.81198687797805e}^{-2}y_{23}-49.2361978412\;$$

(A24) $$x_{24}=3{.44292642962884e}^{-4}y_{24}{}^{2}-1{.95088525902766e}^{-2}y_{24}-149.6236465132\;$$

(A25) $$x_{25}=8{.19803700326146e}^{-4}y_{25}{}^{2}-2{.48594132359937e}^{-1}y_{25}+794.5774447065\;$$

(A26) $$x_{26}=3{.75154707466548e}^{-4}y_{26}{}^{2}-1{.52832756066449e}^{-1}y_{26}-138.7228401308$$

Equations (A27)–(A52):

(A27) $$y_{1}=-8{.4500892281e}^{-3}x_{1}{}^{2}+16.94088086027x_{1}-8104.997895661$$

(A28) $$y_{2}=-6{.393867991146e}^{-3}x_{2}{}^{2}+13.43513064487x_{2}-6553.51591621$$

(A29) $$y_{3}=-4{.549512564531e}^{-3}x_{3}{}^{2}+10.01200304615x_{3}-4939.881501419$$

(A30) $$y_{4}=-4{.518244577148e}^{-3}x_{4}{}^{2}+8.952093236062x_{4}-3787.984622645$$

(A31) $$y_{5}=-2{.402507498491e}^{-3}x_{5}{}^{2}+5.610200779061x_{5}-2443.306507477$$

(A32) $$y_{6}=-6{.010233278600e}^{-3}x_{6}{}^{2}+10.81873853981x_{6}-4274.291994271$$

(A33) $$y_{7}=-3{.614497708002e}^{-3}x_{7}{}^{2}+7.073360866266x_{7}-2742.174871950$$

(A34) $$y_{8}=-4{.237069994487e}^{-3}x_{8}{}^{2}+7.706747363717x_{8}-2785.062200705$$

(A35) $$y_{9}=-6{.170966371196e}^{-3}x_{9}{}^{2}+9.576347661311x_{9}-3056.347455449$$

(A36) $$y_{10}=-1{.116740793190e}^{-3}x_{10}{}^{2}+3.066114266387x_{10}-960.5426709476$$

(A37) $$y_{11}=-2{.010675564919e}^{-3}x_{11}{}^{2}+4.401470832127x_{11}-1422.943998352$$

(A38) $$y_{12}=-1{.906314455616e}^{-3}x_{12}{}^{2}+4.171902190955x_{12}-1275.861001848$$

(A39) $$y_{13}=-3{.181035477800e}^{-3}x_{13}{}^{2}+5.601531485740x_{13}-1586.192408171$$

(A40) $$y_{14}=-2{.524957329257e}^{-3}x_{14}{}^{2}+4.507537314563x_{14}-1033.583612067$$

(A41) $$y_{15}=-4{.886967737468e}^{-3}x_{15}{}^{2}+6.244881334510x_{15}-1175.412397577$$

(A42) $$y_{16}=-1{.170515468933e}^{-2}x_{16}{}^{2}+9.873356558673x_{16}-1585.748185160$$

(A43) $$y_{17}=-1{.669077240334e}^{-3}x_{17}{}^{2}+3.310608186704x_{17}-472.8322687479$$

(A44) $$y_{18}=-1{.796822656313e}^{-3}x_{18}{}^{2}+3.093110472883x_{18}-128.5098829008$$

(A45) $$y_{19}=-4{.613783247914e}^{-3}x_{19}{}^{2}+4.820927360987x_{19}-358.8531962554$$

(A46) $$y_{20}=-5{.789077643825e}^{-3}x_{20}{}^{2}+4.845118755650x_{20}-210.6715800810$$

(A47) $$y_{21}=-2{.482603076529e}^{-3}x_{21}{}^{2}+3.016689538945x_{21}+229.2187895223$$

(A48) $$y_{22}=-2{.581428566220e}^{-3}x_{22}{}^{2}+2.762645788154x_{22}+383.7869915917$$

(A49) $$y_{23}=-2{.060238721019e}^{-3}x_{23}{}^{2}+2.530532601516x_{23}+465.5466135950$$

(A50) $$y_{24}=-1{.850361178625e}^{-3}x_{24}{}^{2}+2.093337244263x_{24}+689.6723894220$$

(A51) $$y_{25}=-3{.355245643787e}^{-3}x_{25}{}^{2}+7.611874637896x_{25}-3591.097656766$$

(A52) $$y_{26}=-2{.237616175281e}^{-3}x_{26}{}^{2}+2.035706833294x_{26}+845.4270033110$$

Equations (A53) and (A54):

(A53) $$\{\begin{array}{c} a=3{.055e}^{-10}{\bar{x}}^{2}-7{.183e}^{-11}{\bar{y}}^{2}+2{.223e}^{-10}\bar{x}\bar{y}+1{.069e}^{-7}\bar{x}+1{.097e}^{-7}\bar{y}+2{.965e}^{-4}\\ b=5{.749e}^{-9}{\bar{x}}^{2}-2{.552e}^{-7}{\bar{y}}^{2}-7{.113e}^{-7}\bar{x}\bar{y}+1{.209e}^{-4}\bar{x}+1{.588e}^{-5}\bar{y}+9{.857e}^{-2}\\ c=-4{.636e}^{-5}{\bar{x}}^{2}+3{.324e}^{-5}{\bar{y}}^{2}+4{.137e}^{-5}\bar{x}\bar{y}+1.023\bar{x}-0.247\bar{y}+9.633 \end{array}$$

(A54) $$\{\begin{array}{c} a\prime =-5{.034e}^{-9}{\bar{x}}^{2}-1{.285e}^{-8}{\bar{y}}^{2}-5{.001e}^{-11}\bar{x}\bar{y}+4{.153e}^{-6}\bar{x}+1{.839e}^{-5}\bar{y}-8{.775e}^{-3}\\ b\prime =8{.300e}^{-6}{\bar{x}}^{2}+1{.222e}^{-5}{\bar{y}}^{2}-1{.277e}^{-5}\bar{x}\bar{y}+4{.970e}^{-3}\bar{x}-1{.503e}^{-2}\bar{y}+6.963\\ c\prime =-5{.794e}^{-3}{\bar{x}}^{2}-3{.412e}^{-3}{\bar{y}}^{2}+8{.521e}^{-3}\bar{x}\bar{y}-4.190\bar{x}+3.772\bar{y}-469.338 \end{array}$$
